# Costs and outcomes of improving population health through better social housing: a cohort study and economic analysis

**DOI:** 10.1007/s00038-017-0989-y

**Published:** 2017-06-13

**Authors:** Nathan Bray, Paul Burns, Alice Jones, Eira Winrow, Rhiannon Tudor Edwards

**Affiliations:** 1 Centre for Health Economics and Medicines Evaluation, Bangor University, Ardudwy Hall, Bangor, Gwynedd LL57 2PZ UK; 2 Socially Sustainable Ltd., Sunderland, UK; 3 Alice Jones Impact Consulting Ltd., Nottingham, UK

**Keywords:** Housing, Health economics, Cost analysis, Cost-consequence analysis, Cohort study, Public Health

## Abstract

**Objectives:**

We sought to determine the impact of warmth-related housing improvements on the health, well-being, and quality of life of families living in social housing.

**Methods:**

An historical cohort study design was used. Households were recruited by Gentoo, a social housing contractor in North East England. Recruited households were asked to complete a quality of life, well-being, and health service use questionnaire before receiving housing improvements (new energy-efficient boiler and double-glazing) and again 12 months afterwards.

**Results:**

Data were collected from 228 households. The average intervention cost was £3725. At 12-month post-intervention, a 16% reduction (−£94.79) in household 6-month health service use was found. Statistically significant positive improvements were observed in main tenant and household health status (*p* < 0.001; *p* = 0.009, respectively), main tenant satisfaction with financial situation (*p* = 0.020), number of rooms left unheated per household (*p* < 0.001), frequency of household outpatient appointments (*p* = 0.001), and accident/emergency department attendance (*p* < 0.012).

**Conclusions:**

Warmth-related housing improvements may be a cost-effective means of improving the health of social housing tenants and reducing health service expenditure, particularly in older populations.

## Introduction

### Housing and health

Housing has a major impact on health and well-being. In 2014/2015, an estimated 43,900 United Kingdom (UK) excess winter deaths occurred in the coldest months of the year (December–March) compared to the rest of the year: the highest figure for 15 years [Office for National Statistics (ONS) 2015]. Approximately 36,300 of these deaths occurred amongst people aged 75 and over. Cold homes, particularly those below 16 °C, cause a substantially increased risk of cardiovascular and respiratory conditions (Mason and Roys 2011). Unsurprisingly, respiratory disease was the underlying cause for over a third of the excess winter deaths in 2014/2015 (ONS 2015).

When mitigated by likelihood and severity, cold and damp are two of the most significant housing hazards in the UK (Chartered Institute of Environmental Health 2008). In 2015, a fifth of homes in England failed to meet the Decent Homes Standard; it is estimated that 16% of private rented homes and 12% of housing association homes still have no form of central heating (Department for Communities and Local Government 2017).

The term fuel poverty refers to the inability to keep a home adequately warm due to the cost of energy bills (Hills 2012). Government schemes such as the Affordable Warmth grants (replacing the Warm Front scheme) have been implemented to help low income households increase indoor warmth and energy efficiency. However, in 2014, approximately 2.4 million households in England (about one in ten) were still in fuel poverty (Department of Energy and Climate Change 2016); thus, more work is needed to tackle this issue.

A systematic review of over 100 years’ worth of evidence found that housing improvements, particularly those aimed at improving warmth, can offer a range of health benefits (Thomson et al. 2009). Furthermore, improving housing has wider impacts on society. The NHS spends £2.5 billion a year on conditions and illnesses whose main contributor is poor housing (Friedman 2010). The burden on the NHS and the UK economy as a whole could be reduced if the standard of housing was improved, particularly relating to warmth and energy efficiency.

To date, the use of robust economic evaluation methods in housing intervention studies has been limited (Fenwick et al. 2013); however, evidence indicates that health service and energy cost savings from retrofitted insulation outweigh intervention costs (Chapman et al. 2009), and that retrofitted insulation has a significant effect on self-rated health, wheezing, school/work absence rates, and health service use (Howden-Chapman et al. 2007). Furthermore, improvements to household ventilation and heating systems have been found to be a cost-effective means of improving the health and quality of life of children with moderate-to-severe asthma (Edwards et al. 2011; Woodfine et al. 2011).

### Applying methods of economic evaluation to housing

In the economic evaluation of public health interventions, the National Institute for Health and Clinical Excellence (NICE) Centre for Public Health Excellence recommends cost-consequence analysis and cost–benefit analysis due to the intrinsic difficulties of conducting cost-utility analysis and quality-adjusted life year (QALY) calculation in this context (NICE 2012, 2013). NICE state that the main disadvantage of cost-utility analysis is narrowness of outcomes, specifically the predominant focus on health benefits. Their technical guidance argues that cost-utility methods focus on efficiency and not equity in health interventions, and, therefore, should be conducted as part of a range of other economic evaluation techniques (NICE 2012).

For the purposes of this paper, housing improvements are defined as any major retrofitted changes or modifications to a home that are specifically undertaken to improve warmth, reduce draft, reduce damp/mould, or to improve energy efficiency. Social housing refers to affordable housing managed by local councils.

### Aims

The over-arching aim of this project was to understand the impact that warmth-related housing improvements have on the health, well-being, and quality of life of families living in social housing. Second, we sought to determine the costs and outcomes associated with new warmth-related housing improvements, compared to existing, unmodified social housing. We hypothesised that the health, well-being, and quality of life of tenants would improve after the installation of housing improvements.

## Methods

The project was funded by Gentoo and Nottingham City Homes. Ethical approval was granted by the Bangor University Healthcare and Medical Sciences academic ethics committee (reference: 2014-03-03). An historical cohort study design was utilised. Households were recruited by Gentoo (April–December 2014) as part of their housing retrofit scheme. Gentoo build, retrofit, and manage social housing in the North East of England. Gentoo have delivered core housing management services and maintenance to over 29,000 homes (Gentoo 2013).

### Study population

A purposive sampling frame was used to recruit families living in social housing who had been assessed by Gentoo and were subsequently scheduled to receive housing improvements to increase warmth, heating, energy efficiency, and damp-proofing. In each household, the main tenant acted as a representative for the household. They were asked to complete demographic questions and provide household health service use estimates on behalf of the household. Where possible, all other members of the household completed a separate health status measure, with main tenants proxy reporting for children under the age of 11.

Main tenants were given a study pack containing a covering letter, information sheet, and consent form as part of the final assessment for their scheduled housing improvement(s). A Gentoo housing officer was present to take consent and administer the questionnaire. The questionnaire was repeated again 12 months after the housing improvements had been completed. Participants were aware that declining to participate would not affect their entitlement to housing improvements provided by Gentoo.

### Intervention

The intervention consisted of installation of new double-glazed windows to replace single-glazed windows, and installation of a new energy-efficient combi boiler. Participants waited an average of 9 days from the point of recruitment to the start of work on their homes. The exact intervention cost per household was not available; therefore, the mean cost to Gentoo per boiler replacement (£2500) and per double-glazed window (£240) was applied. The mean cost of double-glazing was £1425.62 (SD = £800.70) per household. All households received a boiler replacement; a small minority (*N* = 29) did not receive double-glazing. The mean total cost of the intervention per household was £3725.26 (SD = £1041.48).

### Health service use costs

Health service use was measured for each household, as reported by the main tenant in the modified client service receipt inventory (CSRI) section of the data collection questionnaire. Main tenants were asked to estimate how many times all members of the household had collectively accessed primary care [General practitioner (GP)] and secondary care (hospital outpatient, inpatient and accident/emergency) services in the previous 6 months. All members of the household were encouraged to contribute to the completion of the CSRI questions.

Additional information about reasons for service use and hospital department were not collected; therefore, costs were calculated using the Personal Social Services Research Unit (PSSRU) unit costs document (Curtis 2014) and the national schedule of NHS reference costs (Department of Health 2014).

Mean change in health service use costs was calculated using non-parametric bootstrapping, run on 5000 iterations, to produce 95% confidence intervals around differences (Briggs et al. 2006).

### Outcome measures

The primary outcome measure was self-rated household and main tenant health status, as measured using a visual analogue scale (VAS). This thermometer-like scale requires respondents to rate their health today from 0 to 100 (‘worst imaginable health’ to ‘best imaginable health’). All members of the household were requested to complete a VAS.

Aggregated household health status and service use were calculated to account for the wider familial impacts of the intervention, and to acknowledge that housing interventions affect all members of the household.

Secondary main tenant outcome measures included the EQ-5D-3L validated health-related quality-of-life (HRQoL) measure (EuroQoL Group 1990), which is scored from 0 and 1 (‘death’ to ‘perfect health’) and two measures of well-being: the Short Warwick-Edinburgh Well-being Scale (SWEMWBS) (Stewart-Brown and Janmohamed 2008) and an adapted ONS personal well-being measure (ONS 2013a). The SWEMWBS covers aspects of mental health including positive affect (optimism, cheerfulness, etc.), interpersonal relationships and positive functioning (energy, development, competence, autonomy, etc.) (Tennant et al. 2007). It is scored from 7 to 35 (‘worst mental well-being’ to ‘best mental well-being’). The adapted ONS personal well-being measure focuses on well-being and quality of life relating to life satisfaction, happiness, financial satisfaction, and anxiety. The ONS questions are scored on separate scales from 0 to 10 (‘not at all’ to ‘completely’; reversed for the anxiety question) (ONS 2013a).

As an indicator of fuel poverty, main tenants were also asked to estimate how many rooms they left unheated in their homes due to energy costs, and what percentage of their household income was spent on energy bills (less or more than 10%).

### Sample demographic and baseline characteristics

Demographics details are presented in Table 1. A total of 389 households were recruited at baseline. A dropout rate of 41% (*N* = 161) between baseline and 12-month follow-up resulted in 228 households being included in the analyses. Demographic characteristics of the main tenants lost to follow-up are presented in Table 1. Younger participants and people in employment were more likely to dropout, potentially due to time constraints and work commitments.

**Table 1:** Demographic characteristics of main tenants at baseline (England, 2014/2015)

Demographic	All participants (*N* = 389)		Completed participants (*N* = 228)		Participants lost to follow-up (*N* = 161)	
	*N*	%	*N*	%	*N*	%
Age category						
25–34	45	11.6	15	6.6	30	18.6
35–44	55	14.1	23	10.1	32	19.9
45–54	59	15.2	36	15.8	23	14.3
55–64	63	16.2	43	18.9	20	12.4
65–74	76	19.5	44	19.3	32	19.9
75+	86	22.1	66	28.9	20	12.4
Did not answer	5	1.3	1	0.4	4	2.5
Gender						
Male	90	23.1	51	22.4	39	24.2
Female	294	75.6	177	77.6	117	72.7
Did not answer	5	1.3	0	0.0	5	3.1
Marital status						
Single	76	19.5	41	18.0	35	21.7
Co-habiting	46	11.8	18	7.9	28	17.4
Married	108	27.8	60	26.3	48	29.8
Separated	16	4.1	7	3.1	9	5.6
Divorced	53	13.6	34	14.9	19	11.8
Widowed	84	21.6	68	29.8	16	9.9
Did not answer	6	1.5	0	0.0	6	3.7
Employment status						
Full time	37	9.5	12	5.3	25	15.5
Part time	50	12.9	22	9.6	28	17.4
Unemployed	52	13.4	20	8.8	32	19.9
Full time parent/carer	39	10.0	22	9.6	17	10.6
Long-term sickness	33	8.5	33	14.5	0	0.0
Retired	171	44.0	119	52.2	52	32.3
Did not answer	7	1.8	0	0.0	7	4.3
Smoking status						
Non/ex-smoker	256	65.8	156	68.4	100	62.1
Less than 20 per day	94	24.2	59	25.9	35	21.7
More than 20 per day	39	10.0	13	5.7	26	16.1
Did not answer	0	0.0	0	0.0	0	0.0
Annual household income (£)						
<5000	43	11.1	12	5.3	31	19.3
5000–15,000	245	63.0	164	71.9	81	50.3
16,000–25,000	57	14.7	41	18.0	16	9.9
26,000–35,000	15	3.9	4	1.8	11	6.8
36,000–50,000	3	0.8	1	0.4	2	1.2
50,000+	1	0.3	1	0.4	0	0.0
Did not answer	25	6.4	5	2.2	20	12.4

From the 228 recruited households retained at follow-up, a total of 473 tenants participated in the study: 228 main tenants and 245 other tenants. On average, each household contained 2.1 people. Full demographic details are presented in Table 1. The mean age of main tenants was 62 (SD = 16), and 47 (SD = 25) for all members of the household. The vast majority of main tenants were female (77.6%; *N* = 177). Household income was almost half the UK national average at the time of recruitment (£28,200: ONS 2013b); 77.2% (*N* = 166) of participating households had an income of less than £15,000 per year. Furthermore, 86.8% (*N* = 177) of households spent 10% or more of their household income on heating and energy bills, an indication that households were at high risk of fuel poverty (Department of Energy and Climate Change 2013a).

Over half of main tenants were retired (52.2%; *N* = 119) and almost a quarter were either unemployed or on long-term sickness absence (23.2%; *N* = 53). High prevalence of chronic illness was observed in the cohort; 56.1% (*N* = 128) of main tenants had arthritis, 24.6% (*N* = 56) had a respiratory illness, and 30.7% (*N* = 70) had a cardiovascular problem. Considering all members of the household, 33.8% (*N* = 160) had arthritis, 15.0% (*N* = 71) had a respiratory illness, and 23.0% (*N* = 109) had a cardiovascular problem.

On average, main tenants rated their health status at 64.9 (SD = 23.7) out of 100 at baseline. The mean household aggregate score was slightly higher at 68.4 (SD = 21.5). The UK population norm is 82.5 (Kind et al. 1999). When adjusted for people living in public/socially rented housing, this population norm falls to 75.2 (75.1 for people aged 45–54 and 70.5 for people aged 55–64), still above the baseline for this cohort.

The mean HRQoL score at baseline for main tenants was 0.69 (SD = 0.27). The UK population norm is 0.86. This norm falls to 0.76 for people living in public/socially rented housing; and 0.67 for people aged 55–64 living in public/socially rented housing (Kind et al. 1999).

The respective mean scores on the ONS happiness and life satisfaction personal well-being measures were 7.6 (SD = 2.3) and 7.5 (SD = 2.4), comparable to population norms of 7.3 and 7.5, respectively (ONS 2014). The mean score for anxiety was 3.1 (SD = 3.5), again comparable to the UK population norm of 3.0.

The mean mental well-being score of main tenants was 28.1 (SD = 5.3) out of 35.

### Analysis of effects

Analyses were carried out using Microsoft Excel and SPSS v22. Paired sample *T* tests were used to compare the mean scores of each measure before and 12 months after the intervention to examine whether there was a significant change in outcomes as a result of the intervention. Paired sample *T* tests are used to detect significant differences in the means of two related groups, for example, before and after and an intervention.

### Main tenant sub-group analyses

Sub-group analyses were carried out to examine whether main tenant demographic characteristics influenced the effectiveness of the intervention. The key sub-groups were defined as gender (male/female), age (<65/≥65), smoking status (non-smoker/current smoker), and fuel poverty risk (≥10% income spent on energy/<10% income spent on energy). Seasonality was tested by comparing the outcomes and health service use of participants who received the intervention in warmer months (April–August, *N* = 154) with participants who received the intervention in colder months (September–December, *N* = 74). For all sub-groups, paired sample *T* tests were used to compare the mean scores for each measure before and 12 months after the intervention. Independent samples *T* tests were also used to compare the mean change scores of comparable sub-groups, for instance, examining if male and female main tenants had significantly different changes in health status at follow-up.

## Results

### Effectiveness of the intervention

A number of statistically significant effects were observed, see Table 2 for full results: household health status improved by 4.8% (*t* (226) = −2.652; *p* = 0.009), main tenant health status improved by 7.5% (*t* (226) = −3.564; *p* < 0.001), and main tenant financial satisfaction improved by 6.8% (*t* (220) = −2.340; *p* = 0.020). Statistically significant effects were also observed in service use, with household hospital outpatient attendance (*t* (223) = −3.465; *p* = 0.001) and accident/emergency department attendance (*t* (221) = 2.530; *p* = 0.012) both reducing significantly after the intervention. Furthermore, the number of rooms left unheated per household reduced by a total of 0.73 rooms per household (*t* (221) = 5.973; *p* < 0.001), which equated to 23% of households being able to heat at least one additional room. All other variables exhibited non-significant changes from baseline to follow-up.

**Table 2:** Effectiveness results and statistical significance (England, 2014/2015)

	Number	Baseline mean (SD)	12-month follow-up mean (SD)	Mean change (SD)	Paired samples *T* test (*significant result)
Household health status score^a^	227	68.39 (21.45)	71.64 (20.82)	3.25 (18.45)	*t* (226) = −2.652; *p* = 0.009*
Main tenant (MT) health status^a^	227	64.89 (23.66)	69.74 (22.39)	4.85 (20.49)	*t* (226) = −3.564; *p* < 0.001*
MT health-related quality-of-life score^b^	220	0.694 (0.274)	0.684 (0.314)	−0.010 (0.261)	*t* (219) = 0.583; *p* = 0.561
Rooms left unheated	222	0.82 (1.81)	0.08 (0.46)	−0.73 (1.83)	*t* (221) = 5.973; *p* < 0.001*
MT well-being score^c^	186	28.15 (5.28)	28.60 (5.27)	0.46 (4.44)	*t* (185) = −1.403; *p* = 0.162
MT Life satisfaction score^d^	223	7.58 (2.29)	7.67 (2.16)	0.09 (2.18)	*t* (222) = −0.615; *p* = 0.539
MT happiness score^d^	223	7.47 (2.41)	7.72 (2.16)	0.25 (2.34)	*t* (222) = −1.573; *p* = 0.117
MT anxiety score^e^	222	3.05 (3.49)	2.65 (3.11)	−0.39 (3.85)	*t* (221) = 1.518; *p* = 0.131
MT financial satisfaction score^d^	221	5.28 (2.52)	5.64 (2.21)	0.36 (2.30)	*t* (220) = −2.340; *p* = 0.020*

*MT* main tenant, *SD* standard deviation

* Significant effect at *p* < 0.05 level

^a^Measured using self-rated health status visual analogue scale (VAS), asked to rate health today from 0 to 100

^b^Measured using generic EQ-5D-3L questionnaire measure, scored from 0 (death) to 1 (perfect health) using validated UK value set

^c^Measured using Short Warwick-Edinburgh Well-being Scale, scored from 7 (lowest possible well-being) to 35 (highest possible well-being)

^d^Measured using adapted Office for National Statistics personal well-being questions, scored from 0 (worst possible score) to 10 (best possible score)

^e^Measured using adapted Office for National Statistics personal well-being questions, scored from 0 (lowest possible anxiety) to 10 (highest possible anxiety)

### Main tenant sub-group analyses

Independent samples *T* test results indicated a statistically significant effect of age with regard to change in health status (*t* (224) = 2.490; *p* = 0.013) and anxiety (*t* (219) = −2.059; *p* = 0.041). The ≥65 age group had a significant change in health status (13.1%; *t* (108) = −4.661; *p* < 0.001), while <65 age group exhibited a smaller and non-significant change (2.1%; *t* (116) = −0.714; *p* = 0.476). Likewise, the ≥65 group exhibited a significant improvement in anxiety (33.8%; *t* (105) = 2.635; *p* = 0.010) compared to the <65 s whose anxiety worsened by 3.1%, albeit non-significantly (*t* (114) = −0.265; *p* = 0.791). No further statistically significant differences were found between the mean change scores of comparable sub-groups, although there was some variation in the significance of outcomes for certain sub-groups, see Table 3 for full results.

**Table 3:** Effectiveness results by main tenant sub-group (England, 2014/2015)

	Gender		Age		Smoking status		Fuel poverty		Seasonality	
	Male	Female	<65	≥65	Non-smoker	Smoker	High risk	Low risk	Warm months	Cold months
Main tenant (MT) health status^a^										
BL mean (SD)	63.43 (22.53)	65.31 (24.02)	67.97 (25.17)	61.95 (21.39)	64.28 (23.02)	66.21 (25.09)	64.79 (23.16)	65.61 (23.78)	64.07 (24.04)	66.59 (22.93)
FU mean (SD)	67.65 (21.76)	70.34 (22.59)	69.40 (24.37)	70.05 (20.26)	68.77 (22.21)	71.81 (22.79)	68.11 (22.90)	70.99 (21.40)	69.77 (22.60)	69.66 (22.10)
MC at FU (SD)	4.22 (22.86)	5.03 (19.81)*	1.43 (21.74)	8.10 (18.12)*	4.49 (20.75)*	5.60 (20.03)*	3.32 (21.51)	5.38 (19.64)*	5.70 (20.00)*	3.07 (21.48)
MT health-related quality-of-life score^b^										
BL mean (SD)	0.700 (0.263)	0.694 (0.278)	0.718 (0.295)	0.674 (0.246)	0.687 (0.266)	0.710 (0.292)	0.672 (0.268)	0.733 (0.275)	0.694 (0.276)	0.697 (0.272)
FU mean (SD)	0.694 (0.277)	0.683 (0.325)	0.683 (0.350)	0.688 (0.272)	0.687 (0.298)	0.682 (0.349)	0.674 (0.321)	0.709 (0.317)	0.682 (0.322)	0.692 (0.297)
MC at FU (SD)	−0.006 (0.200)	−0.011 (0.276)	−0.035 (0.263)	0.014 (0.255)	0.000 (0.264)	−0.028 (0.253)	0.002 (0.278)	−0.024 (0.254)	−0.012 (0.272)	−0.005 (0.234)
MT well-being score^c^										
BL mean (SD)	29.10 (4.53)	27.87 (5.47)	27.35 (6.02)	29.13 (4.00)	27.69 (5.50)	29.01 (4.76)	27.36 (5.52)	29.39(4.21)	28.08 (5.55)	28.29 (4.68)
FU mean (SD)	28.62 (4.27)	28.60 (5.54)	27.40 (5.95)	30.10 (3.81)	28.36 (5.64)	29.06 (4.47)	27.70 (5.71)	30.02 (4.22)	28.38 (5.51)	29.10 (4.68)
MC at FU (SD)	−0.48 (4.93)	0.73 (4.27)*	0.05 (4.93)	0.97 (3.71)*	0.67 (4.75)	0.05 (3.79)	0.34 (4.64)	0.63 (4.16)	0.30 (4.80)	0.81 (3.54)
MT life satisfaction score^d^										
BL mean (SD)	7.64 (2.13)	7.57 (2.34)	7.23 (2.52)	7.94 (1.96)	7.39 (2.42)	7.99 (1.93)	7.33 (2.56)	7.91 (1.92)	7.64 (2.22)	7.46 (2.44)
FU mean (SD)	7.22 (2.23)	7.80 (2.12)	7.39 (2.33)	7.95 (1.92)	7.58 (2.18)	7.86 (2.10)	7.53(2.23)	7.90 (2.14)	7.61 (2.19)	7.80 (2.09)
MC at FU (SD)	−0.42 (2.36)	0.23 (2.11)	0.16 (2.33)	0.01 (2.02)	0.19 (2.29)	−0.13 (1.93)	0.20 (2.33)	−0.01 (2.10)	−0.03 (2.16)	0.34 (2.20)
MT happiness score^d^										
BL mean (SD)	7.30 (2.51)	7.52 (2.38)	7.04 (2.56)	7.91 (2.16)	7.28 (2.48)	7.88 (2.19)	7.13 (2.67)	7.91 (2.01)	7.44 (2.36)	7.54 (2.51)
FU mean (SD)	7.20 (2.36)	7.87 (2.08)	7.42 (2.38)	8.01 (1.86)	7.61 (2.21)	7.94 (2.05)	7.56 (2.17)	7.93 (2.21)	7.69 (2.15)	7.76 (2.19)
MC at FU (SD)	−0.10 (2.77)	0.35 (2.20)*	0.38 (2.51)	0.10 (2.15)	0.33 (2.54)	0.06 (1.86)	0.43 (2.45)	0.02 (2.31)	0.25 (2.33)	0.22 (2.39)
MT anxiety score^e^										
BL mean (SD)	3.35 (3.62)	2.96 (3.46)	3.26 (3.77)	2.84 (3.17)	3.38 (3.64)	2.32 (3.05)	3.23 (3.69)	2.66 (3.26)	2.61 (3.32)	3.94 (3.69)
FU mean (SD)	2.71 (3.01)	2.64 (3.14)	3.36 (3.36)	1.88 (2.62)	2.56 (3.02)	2.86 (3.30)	3.24 (3.18)	2.06 (2.84)	2.75 (3.20)	2.44 (2.92)
MC at FU (SD)	−0.64 (4.32)	−0.32 (3.71)	0.10 (3.87)	−0.96 (3.76)*	−0.82 (3.96)*	0.54 (3.44)	0.01 (3.86)	−0.60 (3.86)	0.14 (3.75)	−1.50 (3.84)*
MT financial satisfaction score^d^										
BL mean (SD)	5.34 (2.88)	5.26 (2.41)	4.46 (2.64)	6.20 (2.01)	5.11 (2.59)	5.66 (2.33)	4.49 (2.40)	6.12 (2.31)	5.35 (2.59)	5.13 (2.36)
FU mean (SD)	6.00 (2.13)	5.54 (2.23)	4.83 (2.05)	6.56 (2.03)	5.53 (2.13)	5.89 (2.39)	5.04 (2.19)	6.26 (2.13)	5.55 (2.13)	5.83 (2.39)
MC at FU (SD)	0.66 (2.16)*	0.28 (2.34)	0.37 (2.63)	0.36 (1.87)	0.42 (2.26)*	0.23 (2.40)	0.55 (2.35)*	0.14 (2.20)	0.20 (2.29)	0.70 (2.30)*

*Warm months* Participant sub-group who received intervention between April and August

*Cold months* Participant sub-group who received intervention between September and December

*MT* main tenant, *BL* baseline, *FU* 12-month follow-up, *MC* mean change, *SD* standard deviation

* Significant effect at *p* < 0.05 level

^a^Measured using self-rated health status visual analogue scale (VAS), asked to rate health today from 0 to 100

^b^Measured using generic EQ-5D-3L questionnaire measure, scored from 0 (death) to 1 (perfect health) using validated UK value set

^c^Measured using Short Warwick-Edinburgh Well-being Scale, scored from 7 (lowest possible well-being) to 35 (highest possible well-being)

^d^Measured using adapted Office for National Statistics personal well-being questions, scored from 0 (worst possible score) to 10 (best possible score)

^e^Measured using adapted Office for National Statistics personal well-being questions, scored from 0 (lowest possible anxiety) to 10 (highest possible anxiety

There was only found to be an effect of seasonality on anxiety (*t* (220) = −3.028; *p* = 0.003), see Table 3; participants receiving the intervention in colder months exhibited a significant improvement in anxiety (38.1% decrease in anxiety; *t* (71) = 3.313; *p* = 0.001), while participants receiving the intervention in warmer months exhibited a non-significant worsening of anxiety (5.4% increase in anxiety; *t* (149) = −0.458; *p* = 0.648).

### Health service use costs

Results from the cost-consequence analysis are disaggregated by cost and outcome, see Tables 2 and 4 for a full breakdown of outcomes and costs.

**Table 4:** Change in 6-month household health service use from baseline to 12-month follow-up (England, 2014/2015)

	*N*	Baseline frequency (SD)	Follow-up frequency (SD)	Mean change (SD)	Paired samples *T* test	Baseline cost (SD)	Follow-up cost (SD)	Change in cost	CI for Cost difference
General practitioner	227	6.72 (9.25)	6.07 (7.10)	−0.65 (9.07)	*t* (226) = 1.091; *p* = 0.276	£255.45 (£351.45)	£230.51 (£269.98)	−£24.94	−£84.87 to £30.63
Hospital inpatient	221	0.23 (0.60)	0.22 (0.60)	−0.01 (0.88)	*t* (220) = 0.153; *p* = 0.878	£233.48 (£617.19)	£224.14 (£754.44)	−£9.34	−£133.09 to £116.74
Hospital outpatient	224	0.44 (1.34)	0.14 (0.59)	−0.30 (1.31)	*t* (223) = 3.465; *p* = 0.001*	£56.57 (£171.27)	£17.71 (£76.05)	−£38.86	−£64.57 to −£15.43
Accident/emergency	222	0.40 (1.00)	0.22 (0.73)	−0.18 (1.06)	*t* (221) = 2.530; *p* = 0.012*	£68.18 (£178.82)	£37.19 (£103.24)	−£30.99	−£57.33 to −£3.10
Total	220	–	–	–	–	£598.59 (£927.51)	£503.80 (951.31)	−£94.79	−£273.01 to £85.14

*SD* standard deviation, *CI* two-tailed bootstrapped 95% confidence interval

* Significant effect *p* < 0.05

Full health service use cost estimates were obtained from 220 households. Average 6-month health service use costs were £598.59 (SD = £927.51) at baseline and £503.80 (SD = £951.31) at follow-up per household.

Estimated household health service use over 6 months reduced in all sub categories, see Table 4 for full results: per household, the number of GP visits reduced by 9.7% (0.65 visits per household); hospital outpatient visits reduced by 68.7% (0.30 visits per household); accident and emergency department visits reduced by 45.5% (0.18 visits per household); and inpatient stays reduced by 4.0% (0.01 episodes per household). Per household, this equated to a 15.8% (£94.79; 95% CI −£273.01 to £85.14) reduction in health service use costs over 6 months. There was no indication that seasonality had a significant effect on frequency of health service use, see Table 5.

**Table 5:** Sub-group analysis of the effect of seasonality on 6-month household health service use (England, 2014/2015)

	Warm months	Cold months	Independent samples *T* test
General practitioner			
BL mean (SD)	6.86 (8.73)	6.43 (10.29)	*t* (225) = −0.334; *p* = 0.739
FU mean (SD)	6.35 (7.35)	5.49 (6.58)	
MC at FU (SD) [PS *T* test]	−0.51 (8.19) [*t* (152) = 0.780; *p* = 0.437]	−0.94 (10.70) [*t* (73) = 0.760; *p* = 0.450]	
Hospital inpatient			
BL mean (SD)	0.24 (0.60)	0.20 (0.60)	*t* (219) = 1.420; *p* = 0.157
FU mean (SD)	0.17 (0.49)	0.31 (1.08)	
MC at FU (SD) [PS *T* test]	−0.07 (0.64) [*t* (149) = 1.273; *p* = 0.205]	0.11 (1.24) [*t* (70) = −0.768; *p* = 0.445]	
Hospital outpatient			
BL mean (SD)	0.49 (1.47)	0.33 (1.01)	*t* (222) = 1.627; *p* = 0.105
FU mean (SD)	0.09 (0.35)	0.24 (0.91)	
MC at FU (SD) [PS *T* test]	−0.40 (1.51) [*t* (151) = 3.276; *p* = 0.001]*	−0.09 (0.70) [*t* (71) = 1.187; *p* = 0.239]	
Accident/emergency			
BL mean (SD)	0.42 (1.06)	0.35 (0.87)	*t* (220) = 0.536; *p* = 0.157
FU mean (SD)	0.21 (0.54)	0.22 (0.72)	
MC at FU (SD) [PS *T* test]	−0.21 (1.05) [*t* (149) = 2.423; *p* = 0.017]*	−0.13 (1.10) [*t* (71) = 0.964; *p* = 0.338]	

*Warm months* Participant sub-group who received intervention between April and August

*Cold months* Participant sub-group who received intervention between September and December

*BL* baseline, *FU* 12-month follow-up, *MC* mean change, *SD* standard deviation, *PS T test* paired sample *T* test

* Significant effect at *p* < 0.05 level

## Discussion

The mean cost of the intervention per household was £3725.26 (SD = £1041.48). Reductions in health service use after the intervention equated to a 6-month health service saving of £94.79 per household. Assuming that these cost savings could be maintained, it would take around 20 years to recoup the cost of the intervention through health service savings. This is at odds with the Eurofound report (2016), which concluded that economic and societal costs related to housing improvements could be repaid within 18 months through savings in state funded healthcare and improved social circumstances. This discrepancy is likely due to the specific focus on health service use in this study, as wider economic benefits could be achieved through increased productivity and stimulating economic contribution. Furthermore, the health improvements were small in this study; additional benefits may have been observed with a longer time horizon.

The results indicate that warmth-related housing improvements can be a cost-effective means of improving the health status and personal well-being (especially financial satisfaction) of social housing tenants and increasing the number of rooms heated per house. The HRQoL and mental well-being results are less clear, which may reflect the lack of sensitivity of the measures or the need for a longer time horizon.

There is some evidence that participant age had an impact on the effectiveness of the intervention. The ≥65 age group experienced significant improvements in health status and anxiety, while the <65 age group did not. This demonstrates that targeting warmth-related housing improvements at older populations may be a beneficial strategy, potentially due to the higher prevalence of chronic illness in this age group. No further demographic characteristics significantly influenced the effectiveness of the intervention. The results of the sub-group analyses should be interpreted with caution as the sample size of the overall cohort is too small to make definitive statements about specific sub-groups.

Interestingly, levels of happiness and life satisfaction were slightly above UK norms, and mental well-being results were relatively high. This may have been related to the age of participants, as older people tend to have higher life satisfactions scores (ONS 2014). Financial satisfaction increased significantly during the study period, likely a result of the reduction in households spending 10% or more of their income on energy costs.

Previous evidence has demonstrated that housing improvements can be a cost-effective means of achieving better population health; however, the evidence is often lacking in quality and robustness (Fenwick et al. 2013). Retrofits of ventilation (Woodfine et al. 2011; Hamilton et al. 2015), insulation (Chapman et al. 2009; Howden-Chapman et al. 2007), and new heating systems (Edwards et al. 2011; Woodfine et al. 2011) have all been found to be cost-effective means of improving health, and in some respects, the results from this study support these previous findings.

The Eurofound report explored the social and economic costs of inadequate housing across European Union member states, and surmised that the effect of poor housing becomes more apparent across the life-course (Eurofound 2016). This, in turn, has a consequence for future healthcare spending; thus, the economic savings are also evident in the longer term.

The demographic characteristics of this cohort paint a picture of a socioeconomically deprived sample, suffering from a high prevalence of chronic illness and scoring below UK norms in terms of health status. The government-run Warm Front scheme was introduced in England in 2000 as part of the Home Energy Efficiency Scheme to tackle excess winter deaths related to fuel poverty and low indoor temperatures. The scheme offered means-tested grants to pay for a suite of home improvements such as insulation and boilers (Department of Trade and Industry 2001). Over 2.3 million homes benefited from the Warm Front scheme, with high levels of recipient satisfaction (Department of Energy and Climate Change 2013b) and evidence of improved quality of life and well-being (Gilbertson et al. 2006). In 2013, Warm Front was replaced by Affordable Warmth Grants. With fuel poverty still prevalent in the UK, further government investment is required.

Reductions in service use were most noticeable for outpatient appointments and accident/emergency attendance. Without further descriptive information about health service use, we are only able to speculate about the reasons behind reduced service use. Cold homes increase the risk of falls in the elderly (Department of Health 2007), thus increasing the warmth of homes may have reduced the incidence of falls and thus accident/emergency attendance. Outpatient attendance may have reduced due to less need for recurring appointments related to chronic illnesses exacerbated by cold homes, such as respiratory, cardiovascular, and arthritic conditions. Patients with chronic conditions are more likely to be managed through secondary care than primary care.

### Strength and limitations

A major limitation of this study is the lack of a control group, which inhibited our ability to perform a more robust cost-utility analysis. In retrospect, the effect size for HRQoL was small, negative, and non-significant; therefore, the resultant QALYs would certainly have been outside of the NICE cost per QALY cost-effectiveness threshold (unless the theoretical control group had reductions in HRQoL in the time frame). The lack of cost per QALY estimates is a significant limitation; however, NICE guidance for economic analysis in public health no longer prioritises cost-utility analysis over other forms of economic analysis due to the inherent issues of applying the QALY framework to public health interventions (NICE 2012).

Recruitment and data collection had to be carried out around the pre-planned housing modification work. Therefore, resources and time were relatively limited. To simplify recruitment and data collection processes, main tenants were recruited as representatives for the household and thus were asked to estimate household health service use. There are limitations to estimating household data; the accuracy of health service use estimates may have been affected by household estimation. The validity of aggregating across individuals is debatable. It should be noted that the average number of adults per household was around two, therefore most individuals did not have to make estimates for multiple individuals. Furthermore, all members of the household were encouraged to contribute to health service use estimation.

Issues with seasonality were to some extent dealt with by spreading recruitment evenly across the 9-month recruitment period (April–December 2014) and ensuring that data were collected at roughly the same time of year before and after the intervention. Due to Gentoo’s schedule for housing improvements, it was not possible to recruit participants in January–March. This may have introduced some unavoidable issues relating to seasonality. The seasonality sensitivity analyses demonstrated a possible seasonal influence on anxiety, but no further significant influence on other outcomes or health service use.

### Implications for future research

Due to the relatively simple ‘before and after’ cohort study design, the results of this study should be regarded with some caution as there is potential to overestimate the effect of the intervention without a control group comparator. As such, we see this pragmatic research as a means to support and inform future economic analyses with more robust methods.

This study supports the need to integrate health economics into wider evaluations of housing interventions (Lawson et al. 2013). An appropriate and realistic time horizon is a key priority when developing trials in this context, and a wide perspective lens should be employed which accounts for a variety of costs, benefits, and potential savings (Fenwick et al. 2013).

This study demonstrates a potential lack of sensitivity in the EQ-5D to accurately measure utility gains related to housing improvements. For instance, the health status and well-being measures demonstrated significant improvements to health status and anxiety, yet these were not reflected in terms of utility gains. Likewise, Barton et al. (2007) found that improved housing reduced asthma symptoms but had no significant effect on costs or utility. Barton et al. (2007) suggest that this may be related to the short time horizon and the inclusion of all health service use costs rather than just respiratory-related health service costs. A specific housing or public health approach to QALY calculation could improve the viability of cost-utility analysis in this context.

### Conclusions

In conclusion, the findings presented in this cohort and cost-consequence study demonstrate that retrofitting of new energy-efficient combi boilers and double-glazed windows in social housing may be an effective means of improving health status, anxiety, and ability to heat the home, particularly in older populations. However, the translation of these effects to improved mental well-being and HRQoL is limited. This study will help to inform the design of future economic evaluations relating to health and housing, and highlights the need for robust study design in public health economic evaluations.
